# High Physical Activity Level and the Long-Term Risk of Atrial Fibrillation in Two Swedish Cohorts

**DOI:** 10.3390/geriatrics10030080

**Published:** 2025-06-12

**Authors:** Per Wändell, Malin Enarsson, Tobias Feldreich, Lars Lind, Johan Ärnlöv, Axel Carl Carlsson

**Affiliations:** 1 Department of Neurobiology, Care Sciences and Society, Karolinska Institutet, SE-141 83 Huddinge, Sweden; malin.anna.enarsson@ki.se (M.E.); johan.arnlov@ki.se (J.Ä.); axel.carlsson@ki.se (A.C.C.); 2 Center for Primary Health Care Research, Lund University, SE-205 02 Malmö, Sweden; 3 School of Health and Welfare, Dalarna University, SE-791 88 Falun, Sweden; trf@du.se; 4 Department of Medical Sciences, Faculty of Medicine, Uppsala University, SE-751 05 Uppsala, Sweden; lars.lind@medsci.uu.se; 5 Academic Primary Health Care Centre, Stockholm Region, SE-104 31 Stockholm, Sweden

**Keywords:** physical activity, atrial fibrillation, cardiovascular risk factors, strenuous exercise

## Abstract

Background: Associations between high physical activity (PA) levels and incident atrial fibrillation (AF) is found in some earlier studies. We aim to study the association between levels of PA and AF in two cohorts. Methods: We used data from the Uppsala Longitudinal Study of Adult Men (ULSAM) study, initiated in 1970, included men aged 50 years, with 2202 included in the study. Examinations were reiterated three times, with follow-up after in median 33 years, with 3.8–6.0% on the highest PA level. We also used data from the Prospective Investigation of the Vasculature in Uppsala Seniors (PIVUS; with women 50%); mean age 70 years, baseline 2001–2004, median follow-up 15 years, with 961 included in the study, with 4.8% on the highest PA level. Cox regression analysis with hazard ratios (HRs) was used to study association between PA levels and incident AF, adjusted for CV risk factors: systolic blood pressure, LDL- and HDL-cholesterol, BMI, diabetes, and smoking. Results: Totally, in ULSAM 504 men during 59,958 person-years at risk, and in PIVUS 204 individuals during a follow-up of 11,293 person-years experienced an AF. Neither in ULSAM, PIVUS, nor in the meta-analysis of both cohorts, individuals with the highest PA level showed an increased AF risk, compared to individuals with lowest level of PA. Conclusions: The benefits of PA in community dwelling individuals for its benefits to mental, metabolic, and cardiovascular health should guide public recommendations, rather than a possible risk of AF. Lay Summary: We studied the risk of incident atrial fibrillation at various levels of physical activity in two cohorts and found no statistically significant increased risk after adjusting for cardiovascular risk factors (systolic blood pressure, LDL- and HDL-cholesterol, BMI, diabetes, and smoking).

## 1. Introduction

Atrial fibrillation (AF) is one of the most common heart conditions that is expected to double in the next decades [1]. Factors driving the start and progression of AF are a range of comorbidities and associated risk factors. Moderate physical activity could reduce the risk of AF; however, studies have shown that vigorous exercise is associated with increased risk of AF [1,2]. Incidence of AF has been shown to be increased among athletes showing a 2.5-fold risk compared with non-athlete controls [3]. Studies have suggested that both sedentary behaviour and long-term vigorous exercise may increase the risk of developing AF [3].

Knowledge of physical activity, exercise and the risk of atrial fibrillation is still poorly understood and has low-level evidence [1,2]. There are inconsistencies in previous findings, both in the general population and among athletes, and also whether there are differences between men and women [4]. It has been hypothesized, that mechanisms underlying the increased risk of AF with vigorous exercise is distinctly different from the reduced risk with light-to-moderate physical activity [2,5]. Potential mechanisms to explain the increased risk of AF associated with vigorous exercise, also described as the athlete’s heart, are cardiac adaptations including increased vagal tone with lower resting heart rate, increased stroke volume, with better systolic and diastolic function, modified metabolism and also modified electric characteristics [6]. Even if increased vagal tone with lower heart rate is expected to be heart protective, the increased vagal tone seems to increase the vulnerability to arrythmia within the atria through reduction in the atrial refractory period [7]. Other mechanisms are mentioned in studies of athletes such as atrial remodelling, fibrosis, inflammation, and alterations in autonomic tone [8].

We aimed to study the risk of incident AF in two community-based samples with repeated questionnaires of physical activity levels, and with long follow-up in Swedish registers, with the hypothesis that there is an increased risk of incident AF in individuals with the highest reported levels of physical activity.

## 2. Methods

### 2.1. Study Samples

#### 2.1.1. The Uppsala Longitudinal Study of Adult Men (ULSAM)

The ULSAM study was initiated in 1970, and from 1970 to 1974 a total of 2322 men all aged 50 years and living in the city of Uppsala, Sweden, were investigated (ULSAM, http://www.pubcare.uu.se/ULSAM, accessed on 25 May 2025) [9]. Of those, 82% men accepted to participate. This cohort has since then been reinvestigated at ages 60, 70, and 77 years.

#### 2.1.2. The Prospective Investigation of the Vasculature in Uppsala Seniors (PIVUS)

The PIVUS study was initiated in 2001, with participant inclusion completed in 2004. All 70-year old men and women living in Uppsala, Sweden, during this period were eligible for the study and were invited by letter in a randomized order (described in detail on https://www.uu.se/en/department/medical-sciences/research/epidemiological-studies/pivus, accessed on 24 April 2024) [10]. Of 2025 invited individuals, 1016 agreed to participate (52% women) and 929 were included in the analyses. At age 75 the sample was reinvestigated (2006–2009), where 827 participated.

### 2.2. Traditional Risk Factors

The investigations in ULSAM and PIVUS were executed using similar methods, including anthropometrics, blood pressure, blood sampling, and questionnaires regarding socioeconomic status, medical history, smoking habits, medication, and physical activity level [9,10].

The baseline investigation of the ULSAM study, conducted in the early 1970′ies of participants aged 50 years has been described earlier [11]. The most commonly used cardiovascular (CV) risk factors are included in the analysis, i.e., LDL- and HDL-cholesterol, systolic blood pressure (SBP), Body Mass Index (BMI), diabetes, and smoking status. The fasting blood samples were collected in the morning after an overnight fast. Serum levels of cholesterol, triglycerides, and HDL cholesterol were measured by enzymatic techniques, and LDL cholesterol was calculated using Friedewald’s formula. Fasting plasma glucose levels were determined using an oxidase method. Systolic and diastolic blood pressures were measured twice in the right arm twice while participants were in the supine position and after 10 min rest, and the average of the two readings was used. Smoking status was assessed by self-reported questionnaire. BMI was calculated by the rate of weight/squared height (kg/m^2^).

### 2.3. Physical Activity

Leisure time physical activity was assessed by a self-reported questionnaire at each examination and graded from 1 to 4, with only minor differences in the questionnaires used in ULSAM and PIVUS. Participants answered yes to one of four questions best reflecting their activity level: 1. Mainly sedentary behaviour. 2. Walking or cycling for pleasure. 3. Recreational sports or heavy gardening for at least 3 h every week. 4. Being regularly engaged in hard physical training. The questionnaire categories have earlier been validated and also used in other studies [12,13]. In addition, metabolic equivalents (METS), are also given, defined as the amount of oxygen consumed while sitting at rest and is equal to 3.5 mL O_2_ per kg body weight × min [14].

### 2.4. Outcomes

AF ICD-10 code: I48 (atrial fibrillation and flutter); ICD-9 codes: 427.31 (atrial fibrillation), 427.32 (atrial flutter); ICD-8: 427.93 (atrial fibrillation), 427.94 (atrial flutter). There was no loss of follow-up. In ULSAM, the baseline examination was performed from 1970 to 1974, and follow-up in registers was conducted until 31 December 2014, giving four decades of follow-up. In PIVUS, the baseline examination was performed in 2001 to 2004, and follow-up in registers was conducted until on cause-of-death or hospitalizations were obtained to 31 December 2019.

### 2.5. Statistics

The analyses were conducted using Cox proportional hazard models with hazard ratios (HRs) and 95% confidence intervals (95% CIs), and with updated levels of PA and risk factors at 4 occasions in ULSAM (50, 60, 70 and 77 years). Time at risk was calculated from baseline until date of AF endpoint, date of death, or end of follow-up (31 December 2014 for ULSAM and 31 December 2019 for PIVUS), whichever occurred first. Proportional hazard assumptions were checked and were found to be satisfactory. PA was used as a categorical variable with the sedentary group as referent and the other groups being compared to that referent. In ULSAM we also added time-updated registrations on PA levels and the mentioned CV risk factors, i.e., systolic blood pressure, LDL- and HDL-cholesterol, BMI, diabetes, and smoking in the models. In PIVUS we only used baseline values. We also used a model to study how much the PA levels added to the identification of AF obtained by the mostly used CV risk factors by using logistic regression and the area under the receiver operating curve (ROC), as an indication of the predictive value of PA levels, when added to the model with established CV risk factors.

Finally, we performed a random effect inverse-variance weighted meta-analysis of ULSAM and PIVUS on the risk of AF for the various levels of PA.

## 3. Results

### 3.1. General Results

Baseline data of ULSAM and PIVUS are presented in Table 1 and Table 2, with the PA levels in ULSAM and PIVUS shown in Table 3. In ULSAM, the 2202 men with PA levels available at baseline generated 5553 records during a median follow-up of 27 years (maximum 44 years follow-up) at 50, 60, 70 and 77 years of age. During the follow-up period, i.e., with 59,958 person–years at risk, 504 men experienced a first-time AF. In PIVUS, the 961 participants with PA levels available at baseline, and with a total of 11,293 person–years at risk, 205 individuals experienced a first-time AF.

### 3.2. Risk of AF at Different PA Levels

Table 4 shows the relative risk expressed as HRs of incident AF at different PA levels in ULSAM and PIVUS with adjustment for age, with no statistically significant results. In Table 5, the meta-analysis for the combined results of ULSAM and PIVUS at different PA levels for the risk of incident AF is shown. A higher risk of incident AF was found in unadjusted HRs for PA levels 2 and 3 but not for level 4, while no statistically significant increased risk was found in the adjusted HRs. However, the HRs for incident AF for PA levels 2 and 3 showed values close to the statistically significant level, i.e., with the lower bounds of the 95% CI levels of 0.98 and 0.99, respectively.

Adjustments made for LDL and HDL cholesterol, systolic blood pressure (SBP), Body Mass Index (BMI), diabetes, and smoking status.

## 4. Discussion

The main findings of the present study with a long-term follow-up and with updated variables from two community-based cohorts was that higher PA levels were neither associated with a statistically lower nor higher risk of incident AF. However, the meta-analysis showed close to statistically significant higher risk for PA levels 2 and 3.

It is common knowledge that higher PA levels are shown to be associated with lower risk of CVD [15], which has been shown in the ULSAM cohort that we used in the present study [11]. Among the elderly, clear health gains have been shown with higher PA levels [16]. In a review of preventive pathways of healthy ageing, one of the cornerstones for this is listed as tailored PA [17]. On the other hand, AF increase in prevalence with higher age [18].

Regarding the risk of AF, conflicting results regarding the association between PA level and incident AF have been found in some studies [5]. An earlier review found higher PA levels to be associated with lower AF risk, however only among women [4], while other reviews have showed both J- and U-shaped risk patterns; associations between sedentary behaviour as well as vigorous PA, higher risk of AF, and lower risk of AF with moderate PA [2,19]. Yet, among elderly individuals in the cardiovascular health study, light to moderate PA levels were associated with a lower AF risk, while the highest PA level was not significantly associated with any risk of AF [20,21]. The elderly have, in general, lower PA levels than younger individuals, while the AF risk increases with age, and the possible excess risk of incident AF of higher PA levels could be expected to decrease. One study concluded, that engagement in moderate leisure-time PA and regular walking at moderate pace and distance might attenuate the AF risk in a quarter of an elderly population [21]. Another review on the difference between women and men showed divergent findings of vigorous PA and AF; a lower risk of AF among women, but higher among men [22]. Thus, even if the present study showed no statistically significant results, the “close enough” results in the present study connect to the uncertainty regarding the AF risk in different PA levels. The classification of the different PA levels might be too unprecise to discriminate for the risk of incident AF, the high rates in the PA levels 2 and 3, and the low rates in PA levels 1 and 4, in both cohorts could indicate that.

In contrast to the puzzling results regarding the AF risk, in general, in different PA levels, the situation among participants in endurance sports activities seems different, with a shown increased risk [23], while the risk in the general population to some extent seems to be outweighed by the gains of PA. Earlier studies on participants in the Swedish long-distance skiing race in Vasaloppet have found an increased risk among men with higher number of races and among those with faster completion times in the race, but with no significant association present in women [24,25]. Other studies have also shown an increased risk of AF among endurance sports participants [26], and attendants of sport practice [27]. Interestingly, a Norwegian study among women found a non-significantly higher risk of AF present also among women experiencing many years of prolonged endurance exercise [28], but the number of women exposed to prolonged endurance exercise in that study was rather low (9 out of 89 women with ≥40 years of regular endurance exercise were diagnosed with AF). In contrast, a later study on top Swedish endurance athletes found a higher risk of AF among both women and men [29].

There are different possible mechanisms behind the possible association between sports activities and the incident of “lone AF” as described earlier [5,27,30], i.e., without any other CVD or CVD risk factors, that have been proposed, such as “atrial ectopy, increased vagal tone, changes in electrolytes, left atrial dilatation, or fibrosis” [5]. However, a more probable mechanism behind an increased risk of AF in athletes is an increased left atrial size, and a review concluded that “left atrial remodelling in competitive athletes may be regarded as a physiologic adaptation to exercise conditioning, largely without adverse clinical consequences” [31,32]. In special circumstances this might be associated with an increased risk of AF among men [5,24,26,27], yet female endurance athletes may also be affected by the same mechanism [29], even if the opposite is described in some reviews [22]. Animal studies among mice also found that atrial changes, i.e., elevated atrial hypertrophy, fibrosis and inflammation, increased alongside with AF vulnerability [33].

There are limitations of this study. In comparison with other studies, the participants were rather few. The highest PA level (category 4) includes a relatively small proportion of participants in both cohorts, which might limit the statistical power to detect an association in this group. Unfortunately, the assessment of PA into four levels by the questionnaire, gives broad groupings and may lead to misclassifications. Still, the PA categories reflect common patterns of physical activity. One possibility is that the questions neither catch the important advantages nor disadvantages of PA in relation to risk of AF. Another limitation is the probability of residual confounding factors, such as movement problems or chronic obstructive pulmonary disease, which could limit physical activity over time and that we were not able to adjust for. The strengths of the study include a high response rate, careful examinations of participants, and the long follow-up in the National Swedish registers, known to be of high quality [34,35], and with little or no loss to follow-up.

In conclusion, we found no statistically significant association between different PA levels and incident atrial fibrillation. The benefits of PA in community dwelling elderly individuals for mental, metabolic, and cardiovascular health should guide public recommendations, rather than a possible risk of incident of AF. Even if endurance athletes are an exclusive group, the risks of too vigorous PA among elderly could be a reality according to earlier reviews.

## Figures and Tables

**Table 1:** Description of studied variables in ULSAM, a population-based study in Uppsala including men aged 50. Baseline collected from 1970 to 1974, with updated date from five examination cycles.

Examinations	Age 50		Age 60		Age 70		Age 77		Age 82	
	N	Mean (SD)/Proportion	N	Mean (SD)/Proportion	N	Mean (SD)/Proportion	N	Mean (SD)/Proportion	N	Mean (SD)/Proportion
SBP (mmHg)	2291	133.05 (18.01)	1836	142.53 (19.63)	1214	146.77 (18.5)	834	150.52 (19.99)	525	145.06 (17.47)
Triglycerides (mmol/L)	2292	1.93 (1.24)	1836	1.66 (0.7)	1214	1.45 (0.77)	834	1.38 (0.69)	525	1.39 (0.68)
HDL-cholesterol (mmol/L)	2125	1.36 (0.38)	1742	1.28 (0.24)	1213	1.28 (0.35)	832	1.31 (0.32)	524	1.2 (0.29)
LDL-cholesterol (mmol/L)	2123	5.26 (1.19)	1740	4.43 (0.66)	1213	3.89 (0.9)	832	3.47 (0.85)	524	3.39 (0.84)
BMI (kg/m^2^)	2292	25 (3.19)	1836	25.48 (3.28)	1214	26.28 (3.42)	834	26.3 (3.44)	525	26.09 (3.43)
Glucose (mmol/L)	2290	5.58 (0.97)	1836	5.58 (1.43)	1214	5.77 (1.47)	834	5.88 (1.38)	525	5.94 (1.24)
Antihypertensive medication (%)	2322	4	1860	19	1210	35	782	42	477	54
Lipid lowering medication (%)	2322	1	1860	6	1151	9	782	17	477	21
Antidiabetic medication (%)	2322	1	1855	2	1151	6	782	9	477	10
MetS (%)	2123	12	1737	12	1145	15	775	15	474	20
MetS components	2123	1.35 (0.97)	1737	1.39 (0.9)	1145	1.52 (0.94)	775	1.57 (0.92)	474	1.71 (0.96)

SBP denotes systolic blood pressure, HDL denotes HDL-cholesterol, LDL denotes LDL-cholesterol, BMI denotes Body Mass Index, MetS denotes metabolic equivalents.

**Table 2:** Baseline characteristics in PIVUS, a population-based study in Uppsala including men and women aged 70. Baseline collected from 2001 to 2004.

Examinations	N	Mean (SD)/Proportion
Sex (% females)	1016	50
SBP (mmHg)	1012	149.63 (22.68)
Triglycerides (mmol/L)	1013	1.28 (0.6)
HDL-cholesterol (mmol/L)	1013	1.51 (0.43)
LDL-cholesterol (mmol/L)	1011	3.38 (0.88)
BMI (kg/m^2^)	1016	27.03 (4.33)
Glucose (mmol/L)	1013	5.34 (1.61)
Antihypertensive medication (%)	1013	31
Lipid lowering medication (%)	1016	15

SBP denotes systolic blood pressure. HDL denotes HDL-cholesterol. LDL denotes LDL-cholesterol. BMI denotes Body Mass Index.

**Table 3:** Physical activity (PA) levels in ULSAM and PIVUS, respectively, at the different ages.

	ULSAM								PIVUS	
PA Level	Age 50		Age 60		Age 70		Age 77		Age 70	
	N	%	N	%	N	%	N	%	N	%
1	324	14.71	176	10.75	43	3.92	63	8.21	113	11.76
2	800	36.33	863	52.72	370	33.70	271	35.33	594	61.81
3	967	43.91	536	32.74	619	56.38	399	52.02	208	21.64
4	111	5.04	62	3.79	66	6.01	34	4.43	46	4.79
Total	2202		1637		1098		767		961	

Leisure time physical activity was assessed by a self-reported questionnaire at each examination and graded from 1 to 4 as follows: 1. Mainly sedentary behaviour. 2. Walking or cycling for pleasure. 3. Recreational sports or heavy gardening for at least 3 h every week. 4. Regularly engage in hard physical training.

**Table 4:** The association between physical activity (PA) level and relative risk of atrial fibrillation (AF) expressed as hazard ratios (HRs) with 95% confidence intervals (95% CI) with adjustments for age in ULSAM and in PIVUS.

	ULSAM			PIVUS		
	HR	95% CI	*p*-Value	HR	95% CI	*p*-Value
PA level 1	1 (ref)			1 (ref)		
PA level 2	1.22	0.84–1.76	0.29	0.74	0.49–1.13	0.16
PA level 3	1.26	0.87–1.81	0.22	0.75	0.46–1.21	0.23
PA level 4	1.21	0.72–2.04	0.48	1.10	0.57–2.13	0.78

Leisure time physical activity was assessed by a self-reported questionnaire at each examination and graded from 1 to 4 as follows: 1. Mainly sedentary behaviour. 2. Walking or cycling for pleasure. 3. Recreational sports or heavy gardening for at least 3 h every week. 4. Regularly engage in hard physical training.

**Table 5:** Meta-analysis of data from ULSAM and PIVUS regarding risk of atrial fibrillation by different physical activity (PA) levels, with hazard ratios (HRs) of unadjusted and adjusted for cardio-vascular risk factors.

	HR	CI (95%)	*p*-Value
PA level 2 unadjusted	1.37	1.06–1.77	0.015
PA level 3 unadjusted	1.39	1.05–1.84	0.021
PA level 4 unadjusted	1.14	0.77–1.68	0.520
PA level 2 adjusted	1.29	0.98–1.70	0.071
PA level 3 adjusted	1.33	0.99–1.79	0.057
PA level 4 adjusted	1.19	0.79–1.80	0.395

Leisure time physical activity was assessed by a self-reported questionnaire at each examination and graded from 1 to 4 as follows: 1. Mainly sedentary behaviour. 2. Walking or cycling for pleasure. 3. Recreational sports or heavy gardening for at least 3 h every week. 4. Regularly engage in hard physical training.

## Data Availability

The ULSAM study is listed in the SND (Swedish National Data Service). Access to data is limited, contact persons for ULSAM are Martin Ingelsson (martin.ingelsson@pubcare.uu.se) and Vilmantas Giedraitis (vilmantas.giedraitis@pubcare.uu.se).

## Supplementary Materials

The supplementary information of informed consent can be downloaded at: https://www.mdpi.com/article/10.3390/geriatrics10030080/s1.
