# Supplementary material for: High Physical Activity Level and the Long-Term Risk of Atrial Fibrillation in Two Swedish Cohorts
Source: Geriatrics (Basel). 2025 Jun 12;10(3):80. doi: 10.3390/geriatrics10030080 (PMC12192470; doi:10.3390/geriatrics10030080)
Supplement: Supplementary file 1 [file geriatrics-10-00080-s001.zip › geriatrics-3563718-supplementary.pdf]

Informerat och frivilligt samtycke

Bästa deltagare i hälsoundersökningen av 50-åriga uppsalamän 1970–73

Du tillfrågas härmed om du är villig att delta i ett forskningsprojekt som syftar till att karaktärisera ärftliga faktorer som kan ha samband med högt blodtryck eller diabetes eller någon annan sjukdom bland de stora folksjukdomarna.

Det är uppenbart att ärftliga faktorer är av betydelse för hur människor ser ut. Variationer i arvsmassan (DNA) spelar troligen en roll i utvecklingen av olika sjukdomar. Vi är intresserade av att studera om DNA ser annorlunda ut hos de som utvecklat t.ex. diabetes, högt blodtryck, eller hjärt- kärlsjukdomar, jämfört med andra som inte har utvecklat dessa sjukdomar. Vi samarbetar i detta avseende med laboratorier inom och utom Sverige. Din identitet är inte känd för någon av de grupper som vi samarbetar med.

Lämnar du ditt medgivande till att delta i studien innebär detta att du samtycker till att en bit vävnad, som opererats bort vid tidigare tillfälle, får användas i denna studie samt i senare studier av liknande karaktär. Både denna och framtida studier förutsätter för att genomföras godkännande av forskningsetikkommittén i Uppsala. Insamlad information kommer att behandlas konfidentiellt och kodas, vilket innebär att de som arbetar med studien inte har någon möjlighet att identifiera de patienter som deltar.

Vi vill undersöka att din medverkan är helt frivillig. Om du ger ditt samtycke till att delta i studien med senare ångrar dig kan du när som helst meddela att du önskar utgå. All insamlad information om dig kommer då att förstöras.

Med vänlig hälsning

Hans Lithell

Hans Lithell, professor
tfn. 018/17 79 74

Medgivande

Undertecknad samtycker härmed till deltagande i forskningsprojektet enligt ovanstående beskrivning samt att den del vävnad som tillvaratagits för diagnostik i samband med tidigare genomförd operation får användas för det uppgivna forskningsändamålet. Jag är medveten om att mitt deltagande i studien är fullt frivilligt och att jag när som helst och utan närmare förklaring kan avbryta mitt deltagande

....
Namnteckning

....
Datum

....
Namnförtydligande
